# Dataset of photographed lightning events attaching to and around the Brixton tower, Johannesburg, South Africa for the 2015-2016 thunderstorm season.

**DOI:** 10.1016/j.dib.2020.105630

**Published:** 2020-04-29

**Authors:** Hugh G.P. Hunt

**Affiliations:** The Johannesburg Lightning Research Laboratory, School of Electrical and Information Engineering, University of Witwatersrand, Johannesburg, South Africa

**Keywords:** Lightning, Ground-truth, Tall tower, Lightning Videos, Labelled Lightning Dataset

## Abstract

This data article describes a dataset of videos of lightning flashes to and around a tall tower (the Brixton tower) in Johannesburg, South Africa. The videos were collected during the 2015-2016 South African thunderstorm season and a total of 3623 .mp4 videos are available in the dataset. Three different cameras were used, two in a similar location and the third at a different location giving a 90 degree perspective. Each video is timestamped and labelled depending on the type of event seen (attachment to the tower, nearby the tower, far from the tower, intracloud etc.). This dataset provides ground-truth, timestamped evidence of lightning events a known location and of differing types and can benefit atmospheric research scientists as well as lightning detection operators, particularly with regards to evaluating detection networks operating in the area. As the dataset contains a significant number of labelled videos, it also of use to pattern or image recognition supervised machine learning techniques and researchers.

Specifications Table

**Table:** 

Subject	Atmospheric Science; Image recognition
Specific subject area	This dataset specifically relates to the study of lightning and characterisation of lightning flashes. It could further be utilised in Lightning Location System studies as well as other weather/thunderstorm tracking measurements.
Type of data	Image/Photographs/Videos of lightning events in Johannesburg, South Africa.
How data were acquired	A motion-triggered 5-30 fps camera was setup to observe the Brixton tower (a tall tower in Johannesburg, South Africa) during the 2015-2016 thunderstorm season.
Data format	Raw – videos were saved in .mp4 format and each frame is time-stamped.
	The videos have a resolution of 640 × 360, 16:9 and are formatted with H.264/MPEG-4 AVC codec. The videos range in size from 90 to 150 kb and have an average bitrate of 95 kbit/s. The complete dataset is 800 MB.
Parameters for data collection	ISpyⓇ software was used to perform the motion capture. The camera was positioned at the University of the Witwatersrand looking West at the Brixton tower. The motion capture was set to trigger if a percentage of pixels greater than 50 % changed in the frame.
Description of data collection	The dataset consists of 3623 .mp4 videos of lightning events to and around the Brixton tower, Johannesburg, South Africa. The videos were obtained by motion capture camera and have subsequently manually watched and labelled. The have an average frame rate of 7.25 fps and average bitrate of 95 kbit/s.
Data source location	Institution: University of the Witwatersrand
	City/Region: Johannesburg, Gauteng
	Country: South Africa
	Latitude: -26.1925°
	Longitude: 28.0068°
Data accessibility	Repository name: Mendeley Data
	Data identification number: 10.17632/44vkdvrn67.1
	Direct URL to data: http://dx.doi.org/10.17632/44vkdvrn67.1
Related research article	H.G.P. Hunt, K.J. Nixon, I.R. Jandrell, W. Schulz, Can we model the statistical distribution of lightning location system errors better?, Electric Power Systems Research Journal, Volume 178, 2020.

## Value of the Data

• This dataset provides ground-truth evidence of the occurrence of lightning events or flashes with a known timestamp, allowing the data to be compared with other measurements;
• The dataset also provides ground-truth evidence of lightning to a known location (the Brixton tower, Johannesburg, South Africa) as well as evidence that a known location was NOT struck by lightning. Ground-truth evidence of different types of lightning (cloud-to-cloud, upward, downward) is also provided;
• This dataset can benefit atmospheric scientists, lightning protection engineers, climatologists and meteorologists, lightning detection network operators, pattern/image recognition and machine learning experts;
• This dataset can be time-correlated with other measurements (electric and magnetic-field measurements) made within the area – particularly by lightning location systems – to provide visual evidence of detections and evaluate such systems performance;
• The dataset also provides a significantly sized labelled dataset, allowing for the training and testing of supervised machine learning image recognition algorithms;

## 1 Data Description

**Fig. 1:**
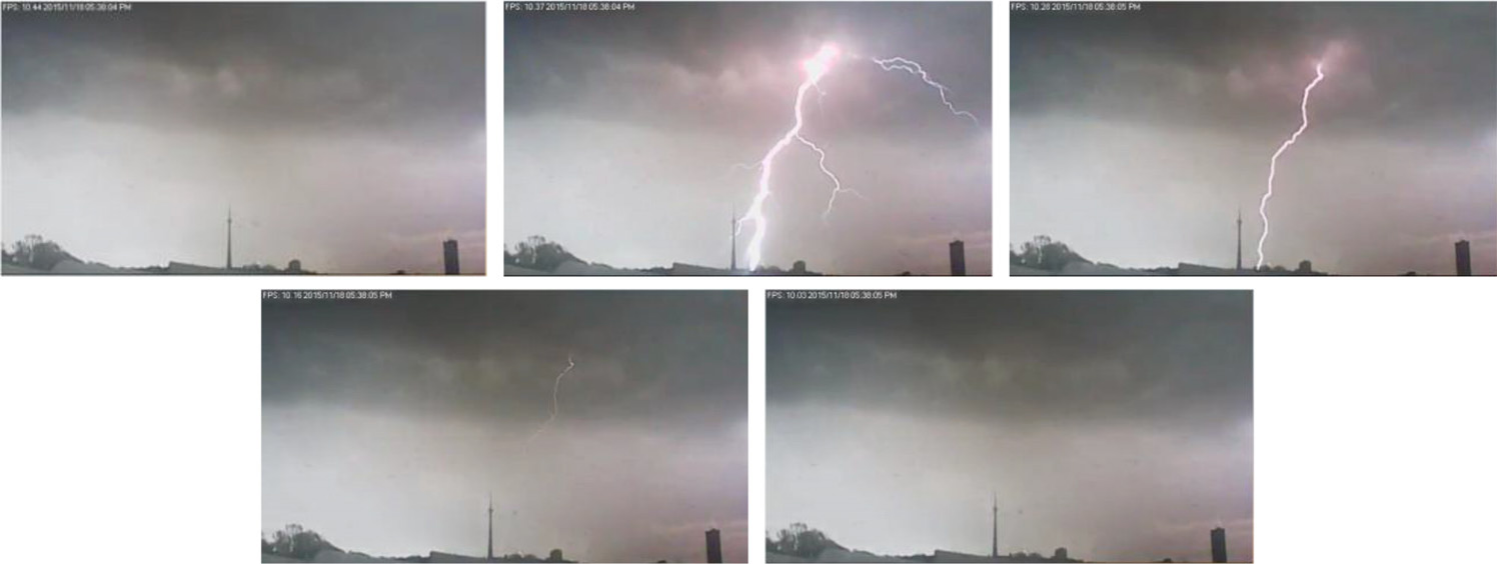
A captured flash sequence indicative of the .mp4 videos contained in the dataset. The flash was captured at 10 fps on 18 November 2015 at 17:38:04 South African Standard time (SAST) (UTC +2).

The dataset described in this article involves lightning events in Johannesburg, South Africa – specifically, to and around the Brixton (also known as the Sentech tower), a tall communications tower just outside the Johannesburg city for the 2015-2016 thunderstorm season [1]. The data files describing these events are: 3623 .mp4 videos of lightning events, Table 1. Description of video labels and Table 2. A list of the GPS timestamps of lightning events to the Brixton/Sentech tower. The lightning events are captured by three different motion triggered cameras operating at approximately 5-30 frames per second with an average frame rate of 7.25 and each video/event is timestamped at South African Standard time (SAST)(UTC +2). The videos are formatted in the H.264/MPEG-4 AVC codec and have a resolution of 640 × 360. 1475 videos were captured by the first camera (Cam 1), 789 by the second camera (Cam 2) and 1359 by the third camera (Cam 3). Fig. 1 shows an example of one of these captures from Cam 1 – a sequence of five frames showing a captured lightning event. In this case, a lightning event attaching to ground, nearby the Brixton tower captured at 17:38:04 SAST on the 18 November 2015.

**Table 1:** Description of labels in file name *YYYY-MM-DD_HH_MM_SS_label_CamX.mp4.*

Label	Description	Example image (Cam 1) Figure 2a-g:	Number of videos
*towerupward*	An upward event initiating from the Brixton tower		Cam 1: 41
			Cam 2: 42
			Cam 3: 26
*towerdownward*	A downward event to the Brixton tower		Cam 1: 9
			Cam 2: 9
			Cam3: 9
*close*	A downward event nearby the tower		Cam 1: 423
			Cam 2: 309
*far*	A downward event far from the tower, in the distance		Cam 1: 125
			Cam 2: 27
*unclear*	It is not possible to see if the flash attached to the ground or was intra-cloud		Cam 1: 269
			Cam 2: 40
*unclearbehind*	No channels were observed, the event was out of the camera field of view		Cam 1: 478
			Cam 2: 336
*CC*	The flash is intra-cloud and did not attach to the ground		Cam 1: 130
			Cam 2: 26

**Table 2:** Lightning flashes to the Brixton/Sentech tower.

Video (type)	Date and Time (SAST)	Date and Time (UTC)
1 (Upward)	14/10/2015 22:05:21	14/10/2015 20:05:21
2 (Upward)	14/10/2015 22:07:10	14/10/2015 20:07:10
3 (Upward)	14/10/2015 22:08:36	14/10/2015 20:08:36
4 (Upward)	14/10/2015 22:10:30	14/10/2015 20:10:30
5 (Downward)	14/10/2015 22:48:19	14/10/2015 20:48:19
6 (Upward)	14/10/2015 22:53:25	14/10/2015 20:53:25
7 (Downward)	14/10/2015 22:57:19	14/10/2015 20:57:19
8 (Upward)	16/11/2015 16:35:08	16/11/2015 14:35:08
9 (Upward)	16/11/2015 18:42:10	16/11/2015 16:42:10
10 (Upward)	16/11/2015 19:34:51	16/11/2015 17:34:51
11 (Upward)	17/11/2015 21:51:53	17/11/2015 19:51:53
12 (Upward)	17/11/2015 21:55:38	17/11/2015 19:55:38
13 (Upward)	17/11/2015 21:58:06	17/11/2015 19:58:06
14 (Downward)	19/11/2015 22:12:54	19/11/2015 20:12:54
15 (Upward)	19/11/2015 22:16:32	19/11/2015 20:16:32
16 (Upward)	19/11/2015 22:38:41	19/11/2015 20:38:41
17 (Upward)	19/11/2015 22:41:16	19/11/2015 20:41:16
18 (Upward)	19/11/2015 22:42:54	19/11/2015 20:42:54
19 (Upward)	19/11/2015 22:45:41	19/11/2015 20:45:41
20 (Upward)	19/11/2015 22:47:12	19/11/2015 20:47:12
21 (Upward)	19/11/2015 22:51:51	19/11/2015 20:51:51
22 (Upward)	19/11/2015 22:56:22	19/11/2015 20:56:22
23 (Upward)	19/11/2015 23:07:58	19/11/2015 21:07:58
24 (Upward)	20/11/2015 21:11:12	20/11/2015 19:11:12
25 (Downward)	20/11/2015 21:14:37	20/11/2015 19:14:37
26 (Upward)	20/11/2015 21:39:40	20/11/2015 19:39:40
27 (Upward)	20/11/2015 21:43:23	20/11/2015 19:43:23
28 (Upward)	20/11/2015 22:17:57	20/11/2015 20:17:57
29 (Downward)	20/11/2015 22:21:58	20/11/2015 20:21:58
30 (Upward)	20/11/2015 22:22:56	20/11/2015 20:22:56
31 (Upward)	20/11/2015 22:24:48	20/11/2015 20:24:48
32 (Upward)	20/11/2015 22:49:50	20/11/2015 20:49:50
33 (Upward)	20/11/2015 22:52:51	20/11/2015 20:52:51
34 (Upward)	20/11/2015 22:54:37	20/11/2015 20:54:37
35 (Upward)	20/11/2015 22:59:55	20/11/2015 20:59:55
36 (Upward)	20/11/2015 23:02:59	20/11/2015 21:02:59
37 (Upward)	20/11/2015 23:43:56	20/11/2015 21:43:56
38 (Upward)	20/11/2015 23:47:40	20/11/2015 21:47:40
39 (Upward)	21/11/2015 22:17:04	21/11/2015 20:17:04
40 (Upward)	02/12/2015 23:48:49	02/12/2015 21:48:49
41 (Upward)	03/12/2015 23:26:30	03/12/2015 21:26:30
42 (Upward)	04/12/2015 23:31:47	04/12/2015 21:31:47
43 (Upward)	04/12/2015 23:33:59	04/12/2015 21:33:59
44 (Upward)	11/12/2015 22:12:14	11/12/2015 20:12:14
45 (Upward)	11/12/2015 22:17:53	11/12/2015 20:17:53
46 (Upward)	27/12/2015 18:58:57	27/12/2015 16:58:57
47 (Upward)	08/01/2016 15:54:38	08/01/2016 13:54:38
48 (Upward)	09/01/2016 18:11:58	09/01/2016 16:11:58
49 (Upward)	09/01/2016 18:15:11	09/01/2016 16:15:11
50 (Upward)	09/01/2016 18:17:50	09/01/2016 16:17:50
51 (Downward)	14/02/2016 19:14:30	14/02/2016 17:14:30
52 (Downward)	14/02/2016 19:17:25	14/02/2016 17:17:25
53 (Downward)	14/02/2016 21:20:48	14/02/2016 19:20:48
54 (Downward)	26/02/2016 20:38:35	26/02/2016 18:38:35
55 (Upward)	26/02/2016 20:43:17	26/02/2016 18:43:17

**Fig. 2:**
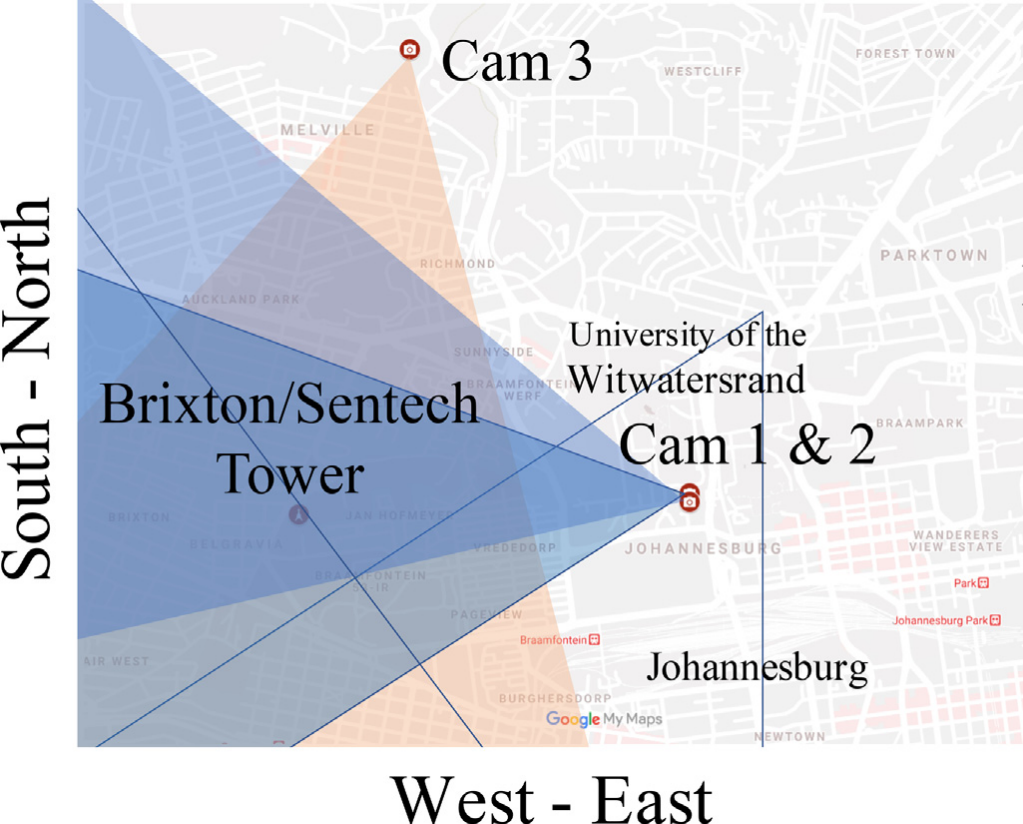
Locations of cameras 1, 2 and 3.

Additionally, to the timestamp, each video is labelled indicating the type of lightning captured in the video. Each file name follows the format *YYYY-MM-DD_HH_MM_SS_label_CamX.mp4.* Table 1 shows the description and an example (figure 2a - g) of each *label* category, as well as the total number videos of this type. Not all Cam 3 files are labelled.

Between Cam 1, 2 and 3 a total of 55 videos of lightning events attaching to the Brixton tower were captured. Table 2 lists these videos and both the upward and downward type are indicated along with the date and timestamp (in both SAST and Universal Coordinated Time UTC). This dataset is used in the associated publication by *Hunt et al.* [2].

## 2 Experimental Design, Materials, and Methods

Johannesburg is the main economic city in South Africa and is located in the Gauteng province in the North of the country. The city itself has two tall communications towers - the Hillbrow tower and the Brixton/Sentech tower. The Brixton/Sentech tower is frequently struck by lightning and is located at 26.1925° South and 28.0068° East.

### 2.1 Experimental Design

**Fig. 3:**
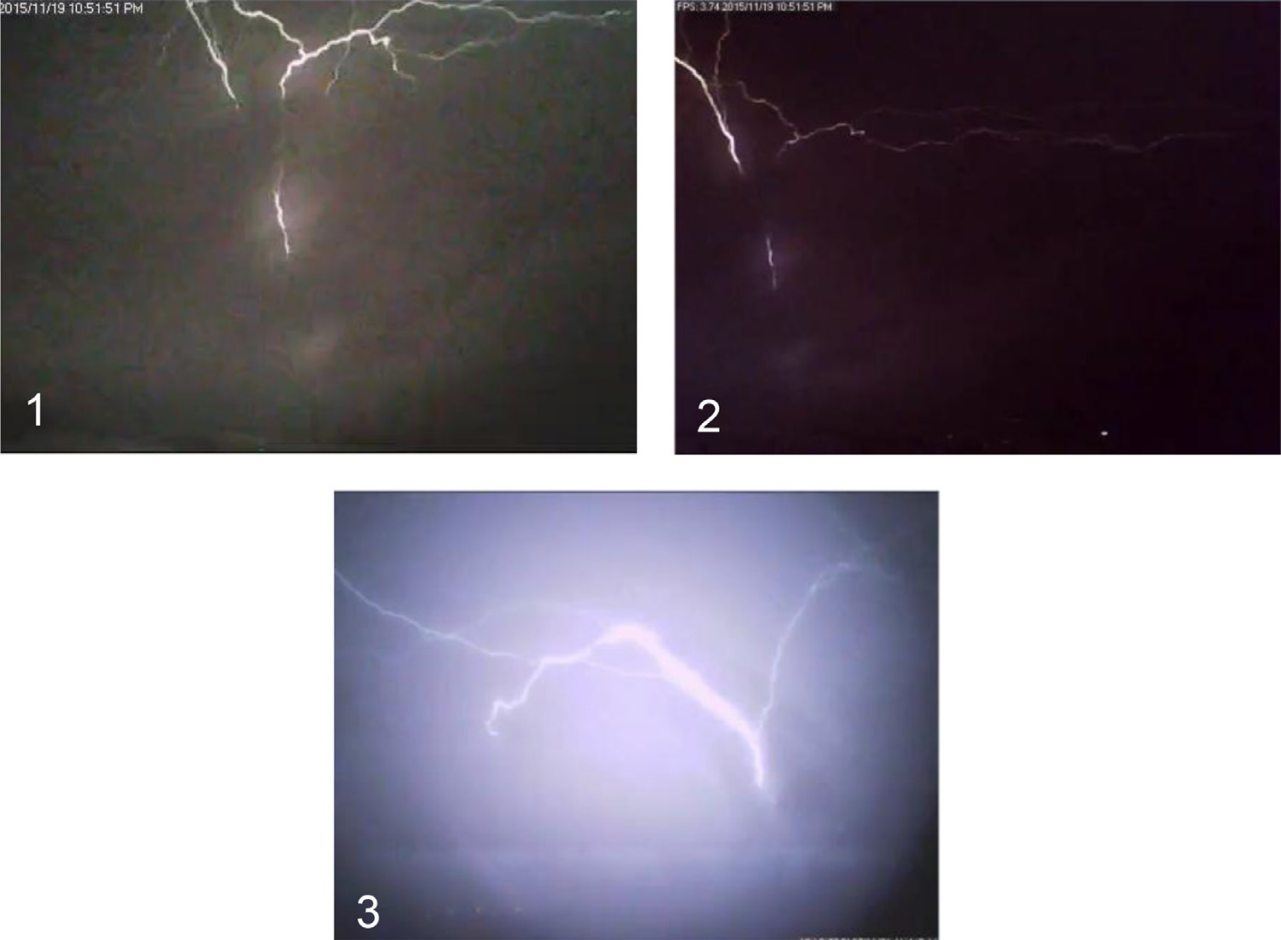
The same lightning flash captured by each of the cameras Cam 1, Cam 2 and Cam 3. Flash 21 from table 2.

Fig. 3 shows the locations of the three cameras and their field of views. Camera 1 and 2 can be found at 26.1914° South and 28.0267° East, approximately 1.88 km East of the Brixton/Sentech tower. Their field of views are both approximately 55° with about 1 km either side of the Brixton/Sentech tower in view and overlap significantly. However, there is a slight offset in angle making for differing perspectives on the footage. The third camera was located North of the Brixton/Sentech tower (26.1727° South, 28.0116° South) and recorded the tower looking South.

**Fig. 4:**
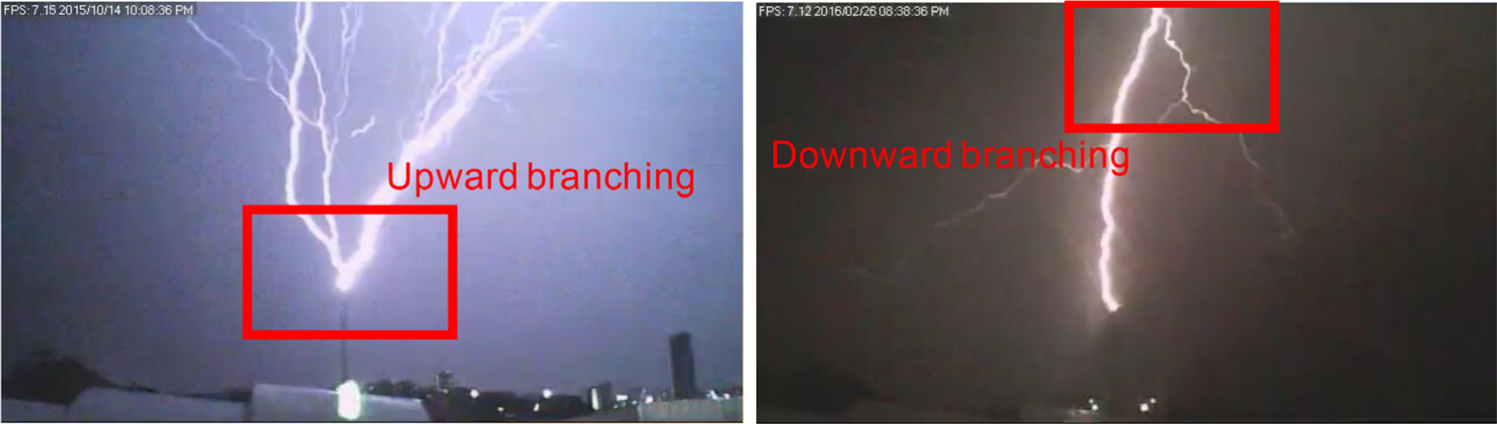
Upward and downward flash illustrating how the two types are distinguished and labelled.
• ***towerdownward***: Downward flashes initiate from the clouds, with multiple channels branching from the clouds until one attaches [3]. Therefore, this label has 2 criteria:
  1. Videos where lightning can clearly be seen attaching to a tall structure (ie. the bright channel ends at the top of the tall tower).
  2. Branching (multiple channels) must begin in the clouds and spread outwards towards the ground, with only one of the branches connecting to the tower (indicated in fig. 5).
• ***close***: Lightning events that do not attach to the tower are downward events (only tall towers initiate upward events) and have channels that extend from the clouds to the ground. The full channel can be seen and if the events were close-by the tower, this should occupy the whole camera frame height. The criteria for this label is therefore:
  1. Videos of lightning events attaching to the ground and NOT the tower.
  2. Videos where the channel extends for 90% of the frame height.
• ***far***: Lightning events that do not attach to the tower are downward events (only tall towers initiate upward events) and have channels that extend from the clouds to the ground. The full channel can be seen and if the events were far from the tower, the channel should be short in length. The criteria for this label is therefore:
  1. Videos of lightning events attaching to the ground and NOT the tower.
  2. Videos where the channel is less than 50% of the frame height.
• ***unclear***: The video is sometimes partially saturated due to brightness of the flash (or rain may obscure clarity) and it is not possible to see the channel. Here, it is not known whether the flash was to ground, upward or in the clouds. Therefore, the criteria for this label is:
  1. Video captures a bright flash, saturating at least 25% of the frame.
  2. No discernible channel can be seen.
• ***unclearbehind***: Lightning events that occur behind the camera are not captured, but the light is reflected in the clouds and this can be sufficient to trigger the cameras. The criteria for these is:
  1. Videos where no channel is visible.
  2. Videos where light reflections can be seen in the clouds.
• ***CC***: Intra-cloud lightning events occur where lightning discharges occur between clouds [3]. In this case, channels can be seen clear but are not vertical and do not extend to the ground. Therefore, the label criteria is:
  1 Videos where a discernible channel can be seen, but is not vertical and does not attach to the ground.

Fig. 4 shows one of the flashes to Brixton/Sentech tower as captured by all 3 cameras. Camera 1 and 2 have a very similar image with a slight perspective shift, but camera 3 shows a significantly different perspective.

### 2.2 Materials

The cameras used were simple webcams and the motion-triggering was performed using iSpy® motion-capture software. The cameras could operate from 5-30 frames-per-second but typically operated between 5-10 frames-per-second (with an average of 7.25 fps) and therefore had an approximate resolution of 60-100 ms and were able to capture numerous images of a lightning flash. However, the resolution was not enough to distinguish the number of individual strokes that constitute a flash. The camera was time-synchronised to the Network Time Protocol (NTP) server of the University of the Witwatersrand.

### 2.3 Methods

During the season, the cameras were checked for captures everyday. Any video captures that were unrelated to lightning (birds flying past the camera, changes in light or cloud cover etc.) were deleted to maintain space while any evidence of thunderstorm activity was kept. Subsequent to the 2015-2016 thunderstorm season, the videos were manually watched and labelled based on the categories described in table 1.

The following criteria is to be used when labelling such datasets:

• ***towerupward*** Upward flashes initiate from tall structures and exhibit branching initiating at the tower tip and splitting into the clouds [3]. Therefore, 2 criteria must be met for this label:
  1. Videos where lightning can clearly be seen attaching to a tall structure (ie. the bright channel ends at the top of the tall tower).
  2. Branching (multiple channels) must begin at the top of the tower and spread outwards towards the clouds (indicated in fig. 5).

## Appendix Supplementary materials

Image, application 1
